# Bone damage and health-related quality of life in Hodgkin lymphoma survivors: closing the gaps

**DOI:** 10.3389/fonc.2024.1201595

**Published:** 2024-02-09

**Authors:** Salvatrice Mancuso, Marta Mattana, Federica Giammancheri, Federica Russello, Melania Carlisi, Marco Santoro, Sergio Siragusa

**Affiliations:** Department of Health Promotion, Mother and Child Care, Internal Medicine and Medical Specialties, Division of Hematology, University of Palermo, Palermo, Italy

**Keywords:** Hodgkin lymphoma, Hodgkin lymphoma survivors, osteoporosis, bone loss, quality of life, osteosarcopenia, ageing, frailty Hodgkin lymphoma

## Abstract

In the recent decades, remarkable successes have been recorded in the treatment of Hodgkin’s lymphoma to the point that today it represents one of the neoplasms with the highest rates of cure and with the highest life expectancy. Nonetheless, this raises the concern for the health of long- term survivors. Late side effects of treatments in synergy with other risk factors expose survivors to increased morbidity and impaired quality of life. In the complexity of the topics concerning these last aspects, an area of growing interest is that of bone damage that follows Hodgkin Lymphoma and its treatments. In this narrative review, we conducted our work through assessment of available evidence focusing on several aspects linking bone damage and quality of life with Hodgkin lymphoma and its treatments. At present, the problem of osteopenia and osteoporosis in Hodgkin lymphoma survivors is a theme for which awareness and knowledge need to be implemented.

## Introduction

We are entering a period of growing interest for cancer survivors due to a number of reasons. In particular, ageing populations and improvements in early diagnosis and treatments have contributed to expand this interest worldwide (1). Within this global context, Hodgkin lymphoma (HL) survivors constitute a particular subset with distinctive features compared to other cancer survivors. HL is divided into two different types, classical HL (cHL) and nodular lymphocyte-predominant HL (NLPHL) (2). The most frequent classical entity is one of the few malignancies for which there is effective therapy, which has translated to over 80% cure rates and a steady decline in mortality (3). cHL has a peculiar epidemiological profile, with a bimodal age distribution, an increasing frequency between the second and third decades, and another peak of incidence after the fifth decade (4). This causes the survivors to be divided into two populations: young adults and older adults. While the increased number of cHL survivors is encouraging, the healthcare needs of this heterogeneous group of subjects are complex and only partially explored. In recent years, research on cHL survivors has focused on two main strands: on one hand, therapy-related long-term toxicity, on the other the Quality of Life (QoL) impairment (5). However, the correlation between QoL and specific late complications has not often been explored. Among the secondary events to cHL and its treatments, bone damage is worthy of attention for the functional consequences that can occur over time and that significantly impact QoL. Here we review the state of research and synthesize the current best available evidence on bone involvement during the trajectory of the clinical history of lymphoma and the effects on QoL, in order to consider preventive strategies and therapeutic interventions.

## Health- related quality of life and late complications in cHL survivors

Health-related QoL (HRQoL) and long-term complications are two closely related topics that encompass all health issues of cHL survivors. The concept of QoL is being largely established in the scientific literature passing through an evolutionary path that has defined the details taken in consideration today (6–8). Driven by increased life expectancy, cultural changes and the enhanced central role of the patient, the QoL is transforming the mission of research and medicine. HRQoL is a multidimensional concept, which describes the psychophysical and the functional well-being according to the patient’s perception. Although HRQoL and QoL are often used interchangeably, in more detail HRQoL namely refers to the implication that health has in the perception of QoL. Late complications have been described in long-term survivors of cHL as a major cause of reduced life expectancy and impaired QoL. The various complications have been related to specific treatments (radiation therapy or chemotherapy) or specific classes of drugs or definite drug or particular regimen. Their recognition has helped to modify antineoplastic therapies over time and to start screening and prevention programs, especially for cardiovascular complications and second neoplasms (9–13). A review of the existing literature reveals that many papers address the challenges of cancer survivors referring globally to different types of cancer, especially non-hematological (14). More space is reserved for cHL as part of papers dealing with long-term survivors of childhood malignancies. Although a useful reference, these publications cannot completely cover all the aspects needed for in-depth knowledge of cHL survivors’ issues (15).

Focusing research on cHL, impairment of HRQoL already appears to be considered a long-term complication rather than the resultant of one or more late toxicity. In this regard, published studies show wide diversification in experimental design, simple size, number of domains of HRQoL and symptoms investigation, type of scales and measures used. Most of these are cross-sectional studies enrolling patients who have completed treatment for at least ten years. Longitudinal studies that began assessment at baseline are poorly represented (16, 17). Available evidence suggests a significant deterioration of the HRQoL in the years following treatment, with prevalent involvement of psychosocial domains (18, 19). Fatigue is the most studied symptom which is reported with significant frequency (20). Fatigue, in turn, correlates with both mental disorders – depression – and late organ complications – mostly cardiopulmonary (21). Unlike other long-term complications, it is not easy to trace the specific causes that determine the impairment of HRQoL.

It is also not clear to identify the predictive factors and recognize the most vulnerable populations, on which preventive intervention can be taken. It is interesting to capture two findings, worthy of being investigated: the role of factors related to the host, age and sex, and the presence of comorbidities. Older age, female sex (22) and the presence of comorbidities tend to negatively affect HRQoL (17). The theme of comorbidities is intertwined with that of ageing. From a biological perspective, cancer and antineoplastic therapies facilitate the aging process, favoring the establishment of a condition of fragility, even in the case of pediatric populations (23, 24). The consequent clinical phenotype can result in a functional impairment that affects the subjective state of well-being.

Great importance is also given to the number of late complications that are observed in cHL survivors. They are considered pathognomonic of the effect of the antineoplastic treatment used. Sterility, neuropathy, cardiomyopathy, coronary artery disease, pulmonary fibrosis, secondary neoplasms and secondary leukemia play a key role in the context of long term- toxicity, with significant effects on survival (25, 26).

As concerns the impact on HRQoL, some repercussions have been described. In young cHL survivors, who were treated with mediastinal irradiation, a variety of cardiovascular abnormalities contribute to alter physical component score of the HRQoL evaluation (27). Observing the temporal evolution of HRQoL, it seems that there is a further worsening of the physical component in relation to the appearance of new cardiopulmonary events (28). It is also very interesting to report the cognitive impairment described in a longitudinal study. The authors highlighted it at the time of diagnosis of cHL, before treatment, and described its further worsening after chemotherapy. No correlation with emotional state was detected, but rather a negative impact on HRQoL (29). Evidence and observations collected thus suggest a synergistic role between lymphoma and chemotherapy on neurological damage, likely based on shared or concomitant biological effects.

## Bone damage in lymphomas

Osteoporosis is a systemic disease of the skeletal system, characterized by low mineral density and deterioration of the micro-architecture of the bone tissue, with a consequent increase in bone fragility (30). This situation leads to an increased risk of fracture due to even minimal trauma. Fragility fractures due to osteoporosis have significant consequences, both in terms of mortality and motor disability, with relevant human, health, economic and social costs. At the origin of osteoporosis is the subversion of the normal bone remodeling process, based on the balance of the RANKL/OPG system, which guarantees an alternation between new bone formation and bone resorption (31). Many factors can alter this balance including antineoplastic chemotherapy. Bone loss and osteoporotic fractures are recognized as adverse events of cancer therapy that may occur already during treatment and as late effects (32). The topic is widely covered in the field of solid tumors, where the causes and potential risk factors are clearly indicated (33). In current recommendations for the prevention and treatment of osteoporosis, lymphomas are counted among the diseases that cause or contribute to osteoporosis and fractures. However, clear indications for prevention and early diagnosis in patients with lymphoma are often not provided, probably due to the attention that the scientific community has been paying to the problem only recently (34, 35).

To gain a better understanding of the importance of bone health and bone damage for cHL survivors, an overview of the topic in lymphomas in general must be considered first. In fact, since both most Non-Hodgkin Lymphoma (NHL) patients and cHL patients share the prospect of achieving long-term remission following first-line treatments and being part of the pool of long-term survivors, a large number of papers published on this field concerns lymphomas as whole. In this context, the importance of long-term toxicity that mainly impacts on HRQoL, rather than on survival, such as second cancers, is increasingly recognized. The low Bone Mineral Density (BMD) and osteoporosis have multifactorial origin and therefore need to be addressed on various levels. At least in part the epidemiology of lymphomas coincides with that of osteoporosis, mainly affecting old age (36, 37). Apart from age, the remaining risk factors already known and included in Fracture Risk Assessment Tool (FRAX) may also be added in patients with lymphoma (38, 39). Already in basal conditions, reduced bone mass has been identified before treatment compared to controls. In a study of 114 patients with non-treated NHL, baseline testing of BMD revealed osteopenia or osteoporosis in 54% of cases (40). In a group of 46 patients with different histologic types of NHL and cHL, with median age of 62 years, 21 (48%) had osteopenia at baseline (41). At the moment there is no clear and definitive explanation to justify this evidence. An abnormal osteoclast differentiation in B-cell malignancies, probably due to differences in the production of local factors acting on bone remodeling, might contribute to this characteristic feature (42–44). A pilot study evaluating 181 patients diagnosed with hematological malignancies showed that both NHL and cHL lymphomas are the group of diseases with the highest percentage of bone loss, 67% and 88% respectively. Chemotherapy treatment could be responsible for this significant prevalence of bone damage in patients with lymphoma in contrast to cases with chronic lymphocytic leukemia (45).

From several studies it becomes increasingly evident that lymphoma therapy acts as a strong causal element for progressive bone loss. In fact, lymphoma survivors are at an increased risk of osteoporosis and subsequent fractures. This evidence derives mainly from observational studies, although it is also supported by prospective studies. Two registry studies involving 8152 and 2589 NHL patients respectively, who received chemotherapy, demonstrated in the treatment group a higher fracture risk compared to the control group (46, 47). Among other observational studies, at least two other retrospective studies in large series of patients treated for DLBCL with R-CHOP demonstrate osteopenia, osteoporosis and increased risk of fractures (48, 49). In an observational study we conducted with smaller sample size, patients in complete remission after first-line treatment for lymphoma, underwent imaging screening for osteopenia and osteoporosis. Almost 50% of cases had signs of osteoporosis and among these 60% had signs of clinically silent vertebral fractures (50). A special group of NHL and cHL survivors is that of patients undergoing autologous or allogeneic bone marrow transplantation that reach high proportions of osteoporosis. The reason for this burden of bone disease is to be found in the different lines of treatment, in the high exposure to glucocorticoids, in the high doses of chemotherapy (51, 52). Other evidence comes from prospective studies. In a group of 32 patients including cases of both NHL and cHL and undergoing first-line therapy, bone density control was evaluated at baseline and after one year, showing significant BMD loss at one year. This study addresses the problem of predictive factors that in this population were numerous, generating obvious difficulties in being able to discriminate the main ones (53). In 61 patients with newly diagnosed NHL, first line chemotherapy was associated to increased bone loss and reduced bone mineral density accompanied by increase of bone resorption markers (54). In a study that employed CT scans to analyze bone loss by measurement of vertebral density in 123 patients with Diffuse Large B-cell Lymphoma (DLBCL) pre- and post-therapy con R-CHOP, substantial vertebral bone loss was documented with a high incidence of fracture. This evidence correlated with two risk factors: low vertebral density at baseline and high International Prognostic Index (IPI). These key factors were associated with higher bone loss and more fracture events as a result of chemotherapy (55).

In light of the numerous evidences that attribute a role to chemotherapy treatment, and in particular to the R-CHOP regimen, in determining bone loss, it is important to question the mechanisms that are at the origin of it. Cyclophosphamide may act indirectly through hypogonadism, causing increased bone resorption. Instead, for doxorubicin, a direct inhibition of bone formation has been invoked (56, 57). Greater attention is devoted to the widespread use of glucocorticoids in different regimens for the treatment of lymphomas at even high doses and as supportive care, which can predominantly affect potential bone damage. The mechanisms of action of glucocorticoids in determining bone loss are multiple and well-known and consist on increased bone resorption, decreased bone formation, calcium retention and endocrinal dysfunction (58). Firmly recognizing the main role of lymphomas chemotherapy in bone damage determination, mostly represented by the use of glucocorticoids, it is necessary to consider all the additional risk factors that can contribute to the deterioration of bone health. Among them: older age, female sex, predisposing history, lymphoma bone involvement at baseline, receipt of prophase steroids (47, 48). Finally, it is worth mentioning the few randomized interventional trials, which have allowed to demonstrate the effectiveness of the prevention of osteopenia induced by chemotherapy and steroids in patients with lymphoma. Both intravenous and oral bisphosphonates were used in the different studies, showing their efficacy in reducing BMD loss and tolerability compared to placebo (59–61). Although the studies under consideration have different designs and evaluate heterogeneous patient populations, altogether they provide a fairly consistent view on the problems of bone loss during the clinical and therapeutic history of lymphomas.

## Bone damage in Hodgkin survivors

Although in recent years researchers and clinicians have explored and brought forward the problem of bone damage in patients treated for lymphoma, only few papers exclusively dedicated to cHL have been published. A recent systematic review of long-term endocrine effects in lymphomas did not capture sufficiently relevant data to suggest targeted follow-up for bone alterations in cHL survivors (62). At the moment it is possible to refer only to a few original articles with experimental retrospective design. Two works from the 90s offer us a glimpse of the effects of previous therapy schemes that had a greater impact on gonadal function. In particular, focusing on the gender of patients, BMD reduction was observed in female with chemotherapy-induced premature ovarian failure (63). In a group of 29 male survivors in remission, treated with MOPP or similar schemes, combined with radiotherapy, after a follow-up of 3 years, significant reduction in BMD was highlighted. This finding was related to hypogonadism secondary to chemotherapy treatment (64). In both works, no other risk factors were identified. Another pair of papers aimed to highlight a possible bone loss in survivors of cHL diagnosed in childhood. In a study group of 109 long-term survivors, with median age at diagnosis of 15.1 years, the proportion of subjects with BMD below the mean did not significantly differed from the general population, in contrast to what is reported in survivors of childhood cancers. One possible explanation for this finding is that the age at which cHL is diagnosed is higher than that of children with Acute Lymphoblastic Leukemia (ALL), when most of the bone mineral content has already been acquired. Older age at diagnosis could mitigate the effects of chemotherapy treatments on bone composition of cHL survivors (65). In the second study focusing on pediatric cHL survivors, a total of 88 subjects treated only with chemotherapy according to MOPP, with a median follow-up of 15.5 years, were evaluated. BMD was decreased only in female participants, probably, as hypothesized by the authors, for premature ovarian failure at adult age as hypothesized by the authors (66).

These studies of pediatric Hodgkin’s, although they trace back to an era when treatments were characterized by higher short- and long-term risks, nevertheless highlight the interrelationship between age at diagnosis, gender, effects on gonadal function and duration of follow-up. In another paper, demonstrating the change from baseline in bone density after standard first line chemotherapy in a retrospective study on 80 patients the role of PET/CT in monitoring any bone damage is underlined. Thus, “opportunistic” assessment by PET is emphasized in screening for osteopenia, considered to be simple and inexpensive, because it is routinely performed in these patients (67). Recently, another group addresses the issue from the point of view of the therapy schemes used in more recent times, and the role of steroids present in the two main protocols, ABVD and BEACOPP, on bone. Assessing mean vertebral density (VDM) changes from baseline, this was demonstrated in 213 patients after chemotherapy treatment, with 14,7% in ABVD group and 20.5% in the BEACOPP group. In multivariate analysis, significant risk factors for prediction of VDM loss were age >30 years of age and chemotherapy protocols other than ABVD 2-4. The study emphasizes the unfavorable role of glucocorticoids, predominantly present both as supportive therapy and as treatment in regimens containing BEACOPP (68). Among the bone problems caused by chemotherapy treatment, the infrequent although significant osteonecrosis (ON) should be mentioned, which can complicate the treatment of hematological malignancies (69). The most consistent study in cHL is that relating to patients enrolled in the German Hodgkin Study Group trials HD10-15 and HD18. Among 11,330 patients, 66 developed symptomatic ON after first-line treatment, 83.3% within three years. The incidence of symptomatic ON was 0.2% in early-stage cHL and 1.0% in advanced-stage cHL. Logistic regression revealed the total cumulative corticosteroid dose to be a strong risk factor interacting with younger age (70). Collectively, data from these studies imply that bone health and bone loss are an important issue in long-term outcomes of cHL survivors.

## Bone damage and health-related QoL in cHL survivors

Bone damage varies along a continuum, with osteoporosis and its consequences in the extreme. Although often asymptomatic, osteoporosis can emerge clinically through three main consequences: pain, fractures and deformity (71). In relation to these cardinal points, osteoporosis becomes one of the main causes of morbidity and mortality, which, in its primary form, affects the elderly and post-menopausal women (72, 73). Osteoporosis compromises the HRQoL of those affected, especially as a result of fractures. In fact, fractures determine disability, institutionalization, hospitalization, limitation of activities, chronic pain and deformities, as well as the risk of death (74). Like any other chronic disease, osteoporosis has significant psychologic and social effects. In fact, it is at the origin of a series of consequences that go beyond the strict physical problems, from anxiety and depression to social withdrawal and isolation. Osteoporosis can transform an autonomous person into a dependent and hopeless subject. Osteoporosis is therefore one of the pathologies of the musculoskeletal system that most compromise the HRQoL (75). It should be noted, however, that the simple reduction of mineral density does not imply a profound deterioration in the QoL. A study has shown a reduction of QoL only in general health perception and mental function domains (76). Individuals with osteoporosis may experience various psychological consequences ranging, at least initially, from generalized anxiety to disease-specific anxiety and finally to depression (77, 78). Conversely, osteoporotic fractures determine decreased physical functioning and symptoms such as pain and fatigue (79). Selecting between the different types and their location, the fractures of the femur and vertebral ones have the greatest impact on the HRQoL for the physical, social and psychological consequences (80). Based on this knowledge, since the osteopenia and osteoporotic fractures represent some of the main health problems for the cancer survivors, we understand how awareness on this topic needs to be expanded. Although it appears implied that the effects of osteoporosis on QoL in cancer survivors are at least similar to those in the general population, data focused on specific survivor populations are lacking. Osteoporosis that develops in the context of a neoplasm and its treatment presents a complex etiology, as the specific oncological risk is added to baseline risk of the general population. In this scenario, it can be imagined that the various causal factors can interact with each other, determining diversified effects on the health and on the QoL. As mentioned, one area susceptible to the effects of osteoporosis is chronic pain. Cancer-induced bone pain has a complex pathophysiology only partially attributable to osteoclast-mediated bone resorption or localization of disease. Neuropathic pain appears constitutive of cancer-induced bone pain through the involvement of mechanisms of central sensitization, neuroinflammation, glial cell activation and an acidic environment. Finally, an etiological distinction needs to be made between neuropathic cancer pain and neuropathic pain in cancer patients: the latter can be caused by cancer treatment and/or comorbidity (81). This intricate crosstalk between pain amplification pathways triggered by cancer can contribute to further impairment of QoL (82). Another area of interrelations is that between bone tissue and muscle tissue, whose alterations act in synergy and with mutual involvement. In recent years, evidence of possible impairment of musculoskeletal health through the coexistence of osteoporosis and sarcopenia has increased. Sarcopenia is characterized by progressive and generalized decline in muscle strength, function and muscle mass with increasing age or secondary to disease (81, 82). This can be at the origin of disability, morbidity, increased mortality, as well as being a predictive factor of fractures (83). Sarcopenia is one of the most typically cancer-related manifestations, as a nutritional marker and prognostic parameter. Its origin is multifactorial, being supported both by the neoplasm and by antineoplastic treatments. Sarcopenia impairs QoL in cancer patients, causing fatigue, inactivity and weight loss (84). The concept of osteosarcopenia was coined on the basis of various evidence that confirmed the simultaneous presence of both pathologies as statistically significant (85–88). In our experience, screening by SARC-F questionnaire showed 62% of cases with sarcopenia (score ≥ 4) in patients with lymphoma observed after remission, who presented a high frequency of bone loss (about 50%) (51, 89). Osteosarcopenia is now thought to have a worse impact on performance and quality of life than isolated osteoporosis and sarcopenia in different clinical settings. From a pathogenetic point of view, it is possible to recognize common pathways that support both conditions, such as genetic polymorphisms of the genes GLYAT, methyltransferase-like 21C (METTL21C), myostatin, α-actinin 3, proliferator-activated receptor gamma coactivator 1-alpha (PGC-1α), and myocyte enhancer factor 2C (MEF-2C).

In addition to the sharing of common pathogenetic mechanisms, a “crosstalk” between bone and muscle has been identified in which fat is the main driver in favoring this interaction. The musculoskeletal unit interacts mechanically and physically but also biochemically via paracrine and endocrine communication Molecular mediators, such as myostatin, may play a simultaneous role in controlling both muscle regeneration and osteoblastic activity (90). Osteosarcopenia as an autonomous nosological entity is, however, a recent field of study, so future developments in the acquisition of new data are expected. Of particular interest will be the impact on the performance status and quality of life of cancer survivors. In cancer survivors with osteosarcopenia, an increase in falls, fractures and disability could be expected that would adversely impact QoL. In addition to genetic factors and lifestyles, ageing in particular plays a decisive role, as demonstrated by the epidemiological profile of osteosarcopenia. Inflammaging, a condition that is progressively established in the life of the individual, characterized by chronic and low-level stimulation of immune system, can determine a structural change of both bone and muscle tissue through different mechanisms. Osteosarcopenia, besides representing a geriatric syndrome, in addition to developing as a consequence of neoplastic disease, is also considered a prognostic factor in at least some solid tumors. Finally, osteosarcopenia contributes to and is associated with the presence of the frailty phenotype (91–93). Osteosarcopenia as an autonomous nosological entity is, however, a recent field of study, so future developments in the acquisition of new data are expected. Of particular interest will be the impact on the performance status and quality of life of cancer survivors. In cancer survivors with osteosarcopenia, an increase in falls, fractures and disability could be expected that would adversely impact QoL.

To summarize, ageing is one of the main drivers that supports the deterioration of bone tissue, individually or in addition to other causal factors such as cancer and its therapies (36). It must be specified, however, that between ageing and cancer there is a mutual boost effect. A theme that shows considerable points of debate and study is that of the acceleration of ageing due to cancer and its treatments (23, 94, 95). Accelerate ageing is a new concept that has developed in the vast field of studies concerning ageing. Aging may proceed on a different trajectory in distinct subjects, based on the interaction of biologic, psychosocial, socioeconomic and environmental factors. Accelerate ageing, which is characterized by biological age more advanced than chronological age, can determine the early appearance and increased severity of age- associated disease. The biological basis of this acceleration consists of the sharing of different hallmarks capabilities between cancer, therapies and aging. In this context, epigenetic ageing together with shortening telomere produces the accumulation of senescent cells The senescent cells, through an inflammation-biased secretome, increase the degradation of nearby tissues and promote the release of inflammatory cytokines, chemokines and damage-associated molecular patterns (DAMPs), which collectively induce the secondary recruitment of inflammatory cells with the further propagation of circulating inflammation and trafficking of immune cells into various tissue compartments. This gradual increase in inflammation impairs the function of several organs and systems, leading to slowing gait speed, declining muscle strength, increasing risks of frailty and an increased risk of comorbidities (such as cardiovascular disease, diabetes or osteoporosis) (96). Accelerated aging may be a candidate mechanism for studying health outcomes and HR- QoL in cancer survivors. Assessment of accelerated aging based on easily measurable biomarkers could serve as modifiable target in bone health interventions.

**Figure 1:**
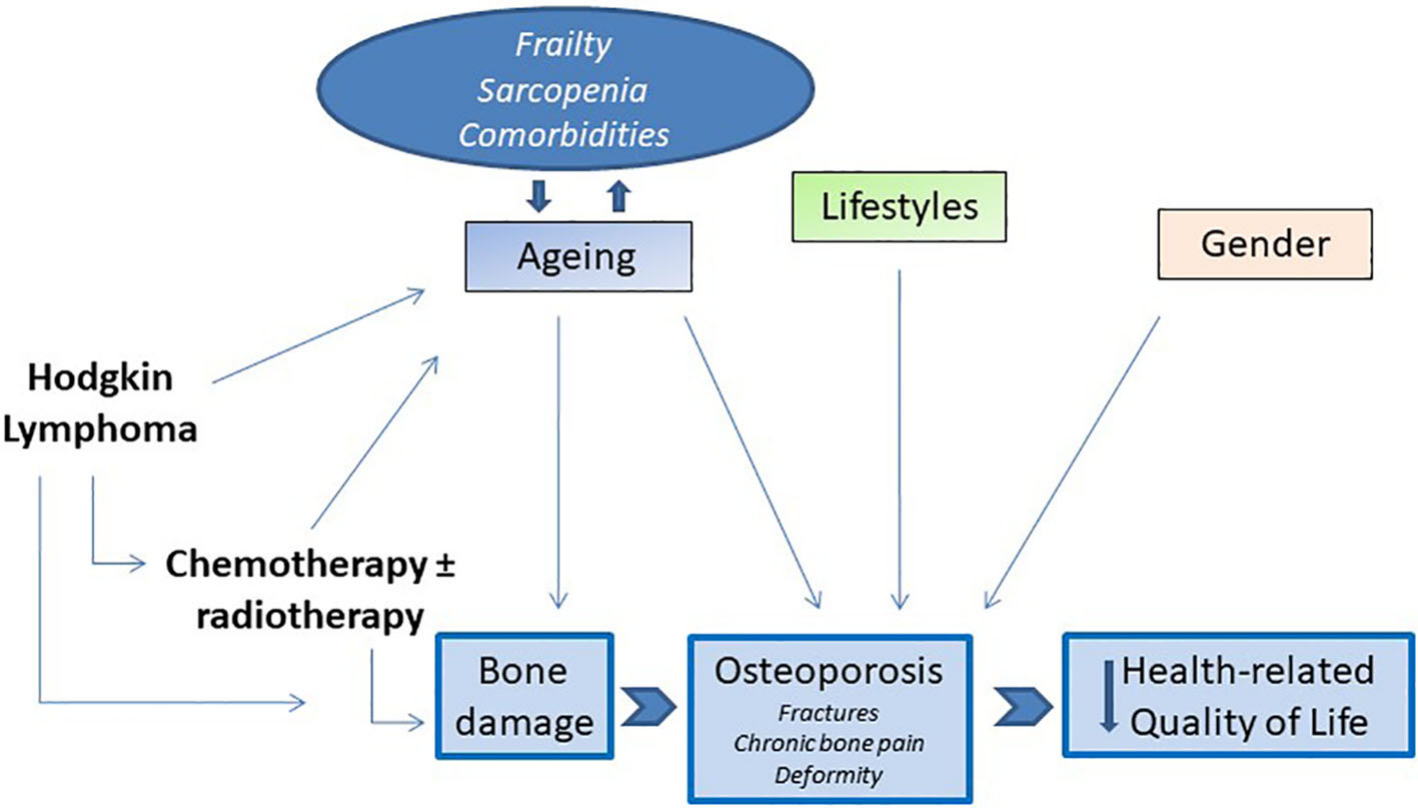
The core hallmarks of interrelationships between bone loss in cHL survivors and impairment of HRQoL.

In light of these considerations, osteoporosis and its consequences can also be considered epiphenomenon of accelerated aging in cancer patients. There is close evidence that cancer survivors have a clinical profile compatible with an accelerated aging phenotype. Greater limitations in carrying out activities of daily living, a greater number of comorbidities, including declining bone health, and more cognitive aging, compared to the population of the same age without cancer history, have been highlighted (97, 98). And finally, probably as a result of all these conditions, the QoL of cancer survivors is compromised compared to the non-cancer population (99–101). Together, these studies demonstrate how osteoporosis is the center of dynamic relationship with multiple regulatory mechanisms aberrantly operative in cancer patients and in cancer survivors (**Figure 1**). These alterations variously contribute to the deterioration of the QoL in the years following treatment and healing. Despite these important premises, the contribution of bone loss and osteoporosis in impacting the QoL of cHL survivors has been understudied. In our pilot study, we gave a group of patients in remission for lymphoma including cHL a disease-targeted tool to measure their quality of life, the mini-OQOL (102). We observed that 55,2% of patients had a moderate score (30-60 points), while the 6.9% had a severe score (<36 points) (50). As already reported, the current information on the impact of bone damage on the health of cHL survivors is sparse, and precisely how the consequences of bone damage compromise the different aspects of their QoL remains to be determined. The principal studies cited in this paragraph are reported in **Table 1** (see).

**Table 1:** Bone damage in classical Hodgkin lymphoma survivors: principal studies cited in the text.

Study	N° of subjects	Risk factors	Time after CHT	Comments
Kreuster ED (63)	49	Women with therapy-induced ovarian failure	2-10 (years, median 5.37)	Regimen: COPP/ABVD +/- irradiation
Holmes SJ (64)	29 (100% male)	Not evidenced	1.1 -6.8 (years, median 3.4)	Possible causes include hypogonadism
Kaste SC (65)	109 (50.5% male)	Males diagnosed at 14 years or older	5.0-12.4 (years, median 7.5)	Median age at diagnosis 15.1 years
van Beek RD (66)	88 (56 M, 32 F)	Women treated with MOPP	5.6-30.2 (years, median 15,5)	None
Ofshenko N (68)	213 (105 female)	Age ≥30 years Escalated BEACOPP Hydrocortisone equivalent doses > 3400 mg/m²	6 months	PET/CT scan used to measure BMD
Borchmann S (70)	11.330 (46.9% female)	Male sex, Total cumulative corticosteroid dose	54.7 (months, median)	The study investigated symptomatic osteonecrosis

COPP, cyclophosphamide, oncovin, procarbazine, prednisone; ABVD, adriamycin, bleomycin, vinblastine, dacarbazine; MOPP, mechlorethamine, oncovin, procarbazine, prednisone; PET, positron emission tomography; CT, computed tomography; BEACOPP, bleomycin, etoposide, adriamycin, cyclophosphamide, oncovin, procarbazine, prednisolone.

## Discussion

After diagnosis and treatment, an entirely new chapter of life commences for cHL survivors, which differs from that of general non-neoplastic population. This new phase must be assessed by some features, such as specific risk factors, possibility of presenting certain side effects generated by previous therapies, increased likelihood of occurrence of age-related diseases. The appearance of a biological and clinical phenotype that characterizes the survivor condition is linked to greater co-morbidities and ultimately mortality, in addition to a non-negligible deterioration in the HRQoL. In this review, our goal was to take into account the most recent publications and latest information about the importance of bone damage in conditioning the cHL survivors to lead a normal and healthy life. Despite the importance of the subject, we can currently recognize a gap in research. Information comes from clinical studies on cancer populations and on lymphoma patients that suggest osteopenia and osteoporosis being direct consequences of chemotherapy and steroid treatment. In addition, iatrogenic events are intertwined with the effects of age, lifestyles and other known risk factors of the general population. Alongside this broad etiology, the role of lymphoma and the accompanying inflammatory state in triggering and sustaining bone destruction should be added. It also necessary to consider how the action of osteoporosis on overall health status and on QoL is equally complex. Several questions remain open: when and how to start the path of monitoring bone health in cHL patients? What strategies to apply to recognize the cases most at risk for bone damage? How to prevent and correct osteopenia and osteoporosis? How to describe the HRQoL and outline its evolution over time in relation to organ impairment and musculoskeletal system in particular? How to integrate the study of bone with geriatric assessment and with the diagnosis of fragility to verify correlations? How to deal with the management of the different comorbidities in a coordinated manner?

The lack of attention in the field of research is also reflected in the limited propensity in the clinical setting to prevent possible bone damage at the beginning of the therapeutic path. It is critical to continue to collect and report on data of bone loss in cHL survivors, thus enhancing evidence quality to inform clinical practice, particularly in an era of rapidly evolving therapies and standard of cares. It is essential that transdisciplinary effort or working group are formed to achieve better results, with perspective and interventional studies. We need to overcome the obstruction of disciplines and make more progress by developing codified multi-specialist monitoring and intervention protocols involving scientific societies. Behavioral interventions targeting host factors, designed to improve physical activity, manage weight and reduce alcohol and tobacco use, can be investigated, both during and after antineoplastic treatment. It is also important that drugs already approved for osteopenia and osteoporosis are part of the regular supportive treatment of the patient with lymphoma during treatment, according to registered indications, after careful focus on bone health. This is to prevent subsequent damage and the worst long-term consequences.

The topic of survivors from a neoplasm with such a high cure rate as cHL raises complex health demands. These requests are addressed to healthcare systems, which in turn are part of the complexity of modern societies. It is important that a “survivorship care plan” that outlines special recommendations for follow-up is offered for Hodgkin survivors. It will probably be necessary to create or implement models of care, based on a multidisciplinary approach involving the family doctor. It must be considered that health is a concept that applies to the person as a whole and not to specific organs. The subjective perception and the degree of awareness of the individual is, therefore, important. In fact, the survivor must also be considered a first-person producer of health states and illness states. In addition to providing adequate health services, it will be necessary to invest in the time dedicated to the doctor-patient relationship and in communication. This is because the correction of lifestyles also depends on individual propensity, education received, available information, socio-economic conditions and access to care (103–106). Finally, cHL survivors must be accurately distinguished from other cancer survivors in order to offer adequate evaluation, treatment and follow-up. The ultimate goal will be not to miss the challenge of improving the quality of life of long-term survivors for cHL implementing strategies and targeted studies focused on bone health.

## Author contributions

SM conception and draft writing, MM wrote sections of the draft, FG and FR reference collection and draft correction, MC and MS draft revision and correction, SS project supervision and final approval. All authors contributed to the article and approved the submitted version.
